# Neonatal mortality among neonates admitted to NICU of Hiwot Fana specialized university hospital, eastern Ethiopia, 2020: a cross-sectional study design

**DOI:** 10.1186/s12887-021-02598-z

**Published:** 2021-03-15

**Authors:** Addis Eyeberu, Hirpo Shore, Tamirat Getachew, Genanaw Atnafe, Merga Dheresa

**Affiliations:** 1 Department of Midwifery, School of Nursing and Midwifery, College of Health and Medical Sciences, Haramaya University, P.O. BOX 138, Dire Dawa, Harar, Ethiopia; 2 Department of Epidemiology, School of Public Health, College of Health and Medical Sciences, Haramaya University, Harar, Ethiopia; 3 Department of Pediatrics, School of Nursing and Midwifery, College of Health and Medical Sciences, Haramaya University, Harar, Ethiopia; 4 School of Nursing and Midwifery, College of Health and Medical Sciences, Haramaya University, Harar, Ethiopia

**Keywords:** Neonatal mortality, NICU, Newborn

## Abstract

**Background:**

In Ethiopia, neonatal mortality is unacceptably high. Despite many efforts made by the government and other partners to reduce neonatal mortality; it has been increasing since 2014. Factors associated with neonatal mortality were explained by different researchers indifferently. There is no clear evidence to identify the magnitude of neonatal mortality and associated factors in the study area. The study aimed to assess the magnitude and factors associated with neonatal mortality.

**Method**s**:**

Facility-based cross-sectional study was conducted among 834 randomly selected neonates. The study was conducted from February 20 to March 21, 2020. Data were extracted from medical records using a checklist adapted from the World Health Organization, and neonatal registration book. The data were inserted into Epi-data version 3.1 and then exported into SPSS window version 20 for analysis. Bivariate and multivariate analyses were employed to identify the association between independent variables and the outcome variable.

**Results:**

Magnitude of neonatal mortality was 14.4% (95% CI:11.9,16.7). Being neonates of mothers whose pregnancy was complicated with antepartum hemorrhage [AOR = 4.13, 95%CI: (1.92,8.85)], born from mothers with current pregnancy complicated with pregnancy-induced hypertension [AOR = 4.41, 95%CI: (1.97,9.86)], neonates of mothers with multiple pregnancy [AOR = 2.87, 95% CI (1.08,7.61)], neonates delivered at the health center [AOR = 5.05, 95%CI: (1.72,14.79)], low birth weight [AOR = 4.01, 95%CI (1.30,12.33)], having perinatal asphyxia [AOR =3.85, 95%CI: (1.83,8.10)], and having early-onset neonatal sepsis [AOR = 3.93, 95%CI: (1.84,8.41)] were factors significantly associated with neonatal mortality.

**Conclusion:**

The proportion of neonatal mortality was relatively in line with other studies but still needs attention. Antepartum hemorrhage, Pregnancy-induced hypertension, place of delivery, low birth weight, having perinatal asphyxia, and having neonatal sepsis were independent factors. The hospital, and health care workers should give attention to neonates admitted to intensive care units by strengthening the quality of care given at neonatal intensive care unit like infection prevention and strengthening early detection and treatment of health problems during Antenatal care visit.

## Introduction

Neonatal death is the death of a newborn in the first 4 weeks of life [33]. Even though there are some variations on causes of neonatal death, the three major causes of neonatal death are infections, prematurity, and birth asphyxia [26].

Globally, 2.5 million neonates died in 2018. Between 2018 and 2030, it is estimated that 27.8 million neonates will die if each country maintains its current rate of reduction in neonatal mortality rate (NMR) [12]. Sub-Saharan Africa had the highest neonatal mortality rate with 28 deaths per 1000 live births in 2018. This is one of the regions with the least progress, accounting for 38% of neonatal deaths in the world. Despite the 18.3% annual reduction rate of the average neonatal mortality from 1990 to 2018 in sub-Saharan Africa, the number of neonatal deaths remains around 1 million deaths per year due to an increasing number of births [12, 27, 28].

In Ethiopia; neonatal mortality was reduced from 39 deaths per 1000 live birth in 2005 to 28 death per 1000 live birth in 2014. However, the current report indicates an increment in neonatal mortality from 28 death per 1000 live birth in 2014 to 30 neonatal death per 1000 live birth in 2019 [6]. More than four-fifth (82.4%) of neonatal deaths occurred within the first week of life [2]**.**

Ethiopia has developed and adopted national and international child health intervention strategies; including a national newborn and child survival strategy (2015–2020) to reduce NMR from 28 to 10/1000 by 2020 [7, 8]. Also, Ethiopia has strived to achieve sustainable development goals (SDGs), aiming to reduce neonatal mortality to at least as low as 12 per 1000 live births by 203 0[34]. Despite this tremendous effort, the reduction of neonatal death is not promising to achieve the intended goal [34].

Risk factors for neonatal mortality such as place of residency, antenatal care (ANC) follow-up, and neonatal illness were indifferently explained by different authors [4, 11, 13, 14, 21, 24]. So, identifying those factors will be important to guide the development of focused and evidence-based health interventions to reduce neonatal mortality. There is no clear evidence found to identify the magnitude of neonatal mortality and associated factors. Therefore, this study aimed at assessing the magnitude and factors associated with neonatal mortality among neonates admitted in a neonatal intensive care unit (NICU) at Hiwot Fana Specialized University Hospital (HFSUH), Harar, Eastern Ethiopia.

## Methods

### Study setting and period

This study was conducted at the neonatal intensive care unit of Hiwot Fana Specialized University Hospital, in Harar City. The city is located 517.2 km towards the east of Addis Ababa, the capital of Ethiopia. HFSUH currently provides different services for approximately 5.8 million people in the catchment area. The NICU is one of the Intensive Care Unit (ICU) services that the hospital is currently running. The hospital admits about 120 neonates per month on average and the unit is divided into a septic room, kangaroo mother care (KMC) room, and critical and subcritical rooms. The unit has 19 neonatal beds and 14 KMC beds, 5 incubators, 10 radiant warmers, and 4 phototherapy machines. Additionally, there are 8 infusers, 4 oxygen cylinders, pulse oximetry, glucometer, and neonatal resuscitation equipment. The unit is staffed with 6 pediatricians, pediatric residents, 4 neonatal nurses, and 7 clinical nurses. The study had been conducted from February 20 to March 21, 2020.

### Study design and population

A facility-based cross-sectional study design was conducted among 834 randomly selected neonates admitted to NICU of HFSUH. All neonates admitted to NICU of HFSUH from December 1, 2016, to December 31, 2019, were the study population. Neonates with incomplete medical records, neonates referred to other places, and left against medical advice were excluded from the study.

### Sampling size determination and sampling procedure

The sample size was calculated by using a single population proportion formula with assumptions of confidence level at 95% = 1.96, a margin of error (d) = 0.03, and a reasonable proportion of neonatal mortality (*P* = 0.23) from a previous study conducted at NICU of Gondar referral hospital [14] and adding 10% non-retrieval rate, the final sample size became 834. The total number of neonates admitted to NICU from December 1, 2016, to December 31, 2019, was 4204. The sampling frame was prepared for those study populations (admitted neonates) using their Medical Registration Number (MRN) obtained from their medical records. Finally, the study subjects that had been included in the study were identified by using a simple random sampling technique (computer-based) from the sampling frame (*N* = 4204).

### Data collection methods and quality assurance

Data were extracted from medical records of newborns using a checklist adapted from the WHO document of review and audit of neonatal death [32] and registration book neonates. We used a pretested checklist containing four sections such as; socio-demographic characteristics related factors; the age of mother and residence; Antenatal related factors such as ANC visit, parity, and type of pregnancies; Neonatal related factors such as neonatal illness, gestational age, birth weight, sex of neonate, APGAR score, and length of stay; Intrapartum related factors such as delivery complications, place of delivery, and mode of delivery. The data were collected by well-trained 10 BSc nurses and supervised for quality of data extraction.

### Operational definitions

Neonatal mortality is defined as the number of neonates who died at the neonatal intensive care unit throughout the study period [20]. Magnitude of neonatal mortality means that the proportion of neonatal death among neonates admitted to NICU.

Cause of neonatal death means that any medical or other causes diagnosed by pediatricians after conducting the necessary laboratory investigation and recorded on the medical chart as a cause of neonatal death*.*

Inadequate Antenatal visit means that having ANC follow-up < 4 times [24].

Hypothermia: Any low body temperature measurement (< 36.5 °C) was diagnosed and recorded on charts during the admission of neonates [17].

Prematurity is described as live-born neonates delivered before 37 completed weeks that are already diagnosed by professionals in charge of the admission of the neonate to neonatal intensive care units [4].

Sepsis: Record of infection or sepsis diagnosed either clinically or with culture by professionals during admission of the neonate and recorded on the chart [17].

Birth asphyxia: is diagnosed whenever a neonate had an Apgar score < 6 in the fifth minute and/or if he/she did not cry immediately after birth; had respiratory distress, floppiness, loss of consciousness, presence of convulsion, and loss of neonatal reflexes [4].

Birth weight is classified using WHO weight classification, Low birth weight is any child with birth weight is less than 2500 g [30]. All other assessments are based on physician judgment as written in the patient card.

### Data processing and analysis

The data were coded, edited, cleaned, and entered into Epi data statistical software version 3.1 and then exported to SPSS version 20 for analysis. A descriptive statistical analysis was used to summarize data. The information was presented using frequencies, tables, and figures. Neonatal mortality was categorized into died (coded 1) and survived (coded 0).

Bivariate analysis and multivariate analysis were done to observe the association between independent variables and the outcome variable by using binary logistic regression. All variables with *P* ≤ 0.25 in the bivariate analysis were included in the final model of multivariate analysis to control all possible confounders. The model goodness of fit was tested by the Hosmer-Lemeshow statistic and Omnibus test. The model was considered a good fit since it is found to be insignificant for Hosmer-Lemeshow statistic (*p* = 0.410) and significant for Omnibus tests (*p* = 0.000) The multi co-linearity test was carried out to observe the correlation between independent variables using VIF, and standard error, no variables were observed with VIF of > 10 and standard error > 2. The direction and strength of statistical association were measured by the odds ratio with 95% CI. The adjusted odds ratio (AOR) along with 95% CI was estimated to identify factors for neonatal mortality by using multivariate analysis in the binary logistic regression. In this study *P*-value < 0.05 was considered to declare a result as a statistically significant association.

## Result

### Socio-demographic characteristics

Out of 834 selected neonates, 2 neonates were referred to Black Lion Hospital, 5 neonates were left against medical advice and 6 neonates had incomplete medical records. A total of 821 neonates’ outcome status was included in the analysis. Out of those, 534 (65%) were from outside of Harar City. Four hundred forty-one (53.7%) neonates were males. Maternal age was documented for 735 (89.5%), of which 51 (6.9%) of the mother were below age 19 and 77.9% of mothers were between 20 and 35 years old.

### Obstetrics characteristics

More than three-fourth (75.2%) of mothers had ANC follow-up. There were 47 (5.7%) mothers with a history of neonatal death. The majority of 751 (91.5%) mothers had a singleton pregnancy. Two hundred fifteen (26.2%) mothers were experienced with obstetric complications during the current pregnancy. The most common complication was Antepartum Hemorrhage (APH) 81(37.7%), followed by pregnancy-induced hypertension 79 (36.7%), premature rupture of membrane (PROM) 47 (21.9%), and preterm labor 29 (13.5%). The majority of 698 (85%) of the neonates were born at the hospital. More than half (69.4%) of neonates were term while 247 (30.1%) of neonates were preterm (Table 1).

**Table 1:** Obstetrics related characteristics of the mothers who gave birth to neonates admitted to HFSUH, Harar Ethiopia, 2020

Variables	Frequency	Percentage
ANC follow-up (*n* = 821)		
Yes	617	75.2
No	204	24.8
Number of ANC visit (*n* = 617)		
≤ Three	468	75.9
Four and above	149	24.1
Parity (*n* = 821)		
Primiparous	315	38.4
Multiparous	388	47.3
Grand multiparous	118	14.4
Type of pregnancy (number of gestations) (*n* = 821)		
Singleton	751	91.5
Multiple	70	8.5
History of neonatal loss(*n* = 821)		
Yes	47	5.7
No	774	94.3
Complication during pregnancy(*n* = 821)		
Yes	215	26.2
No	606	73.8
Place of delivery (*n* = 821)		
Home	35	4.3
Health center	88	10.7
Hospital	698	85
Onset of labor (*n* = 821)		
Spontaneous	742	90.4
Induced	40	4.9
C/S before onset	39	4.8
Duration of labor (*n* = 821)		
< 24 h	778	94.8
≥ 24 h	43	5.2
Mode of delivery (*n* = 821)		
SVD	612	74.5
C/S	175	21.3
Instrumental	34	4.2
Gestational age at delivery (*n* = 821)		
Preterm	247	30.1
Term	570	69.4
Post-term	4	0.5

### Clinical characteristics of neonates at admission

Four hundred eighty-five (59.1%) of neonates were admitted within 24 h of life. The median age at admission was 8 h with an interquartile range of 71 h. The majority of 660 (80.4%) neonates stayed at the hospital for less than 7 days. The median hospital stay was 72 h with an interquartile range of 120 h (Table 2).

**Table 2:** Clinical characteristics of neonates admitted at HFSUH, Harar, Ethiopia, 2020

Variable	Frequency	Percentage
Age of neonate on admission (*n* = 821)		
< 24 h	485	59.1
24–168 h	208	25.3
≥ 168 h	128	15.6
Birth weight recorded (*n* = 716)		
Normal	422	58.9
LBW	217	30.3
VLBW	43	6
EVLBW	2	0.3
Macrosomia	32	4.5
APGAR score on 1st minute (*n* = 674)		
Low	129	19.1
Moderate	285	42.3
Normal/ reassuring	260	38.6
APGAR score on 5th minute (*n* = 674)		
Low	24	3.6
Moderate	198	29.4
Normal /reassuring	452	67.1
Length of stay at hospital (*n* = 821)		
< 7 days	660	80.4
≥ 7 days	161	19.6

**Fig. 1:**
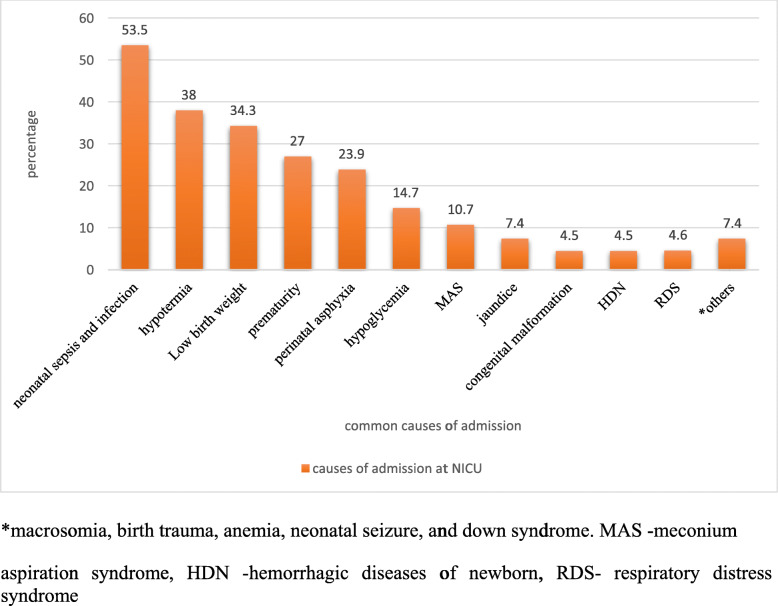
Causes of admission at NICU of HFSUH, Harar Ethiopia

Concerning admission problems, more than half (53.5%) of admissions were due to neonatal sepsis. Of those,62 died, accounting for 52.5% of total neonatal mortality. On the other hand, hypothermia accounts for 312 (38%) of admissions and accounts for 2.5% of neonatal deaths. Low birth weight was the third cause of admission to NICU, 282 (34.3%), and 59 (50%) of neonatal admissions and deaths respectively. There were 5 neonates exposed to HIV (Fig. 1).

### Magnitude of neonatal mortality

**Fig. 2:**
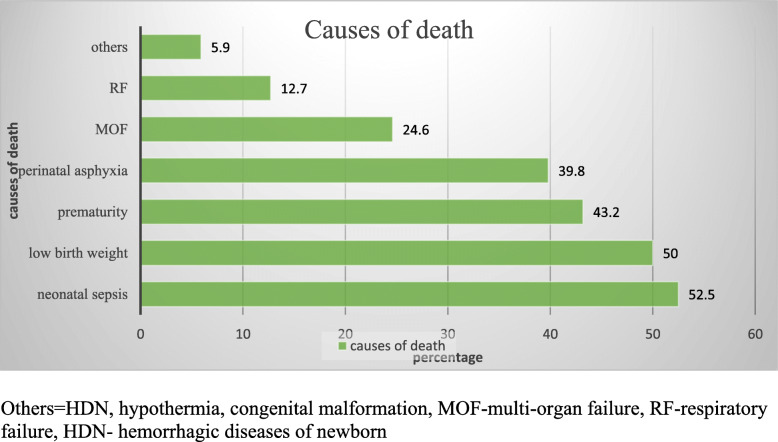
The leading causes of neonatal mortality at HFSUH, Harar, Ethiopia

From a total of 821 neonates admitted over 3 years, 118 neonatal deaths were recorded in the neonatal intensive care unit of Hiwot Fana Specialized University Hospital. Accordingly, the magnitude of neonatal mortality becomes 14.4% (95% CI:11.9,16.7). Neonatal sepsis, low birth weight, and prematurity were identified as the leading cause of neonatal mortality (Fig. 2).

### Factors associated with neonatal mortality

Antepartum hemorrhage, pregnancy-induced hypertension, type of pregnancy, place of delivery, LBW, neonatal sepsis, and Perinatal Asphyxia (PNA) were significantly associated with neonatal mortality.

Multivariate analysis indicated that neonates born from mothers with current pregnancy complicated with antepartum hemorrhage (APH) were 4.13 times [AOR = 4.13, 95%CI (1.92,8.85)] more likely to die as compared to neonates born from mothers without pregnancy complicated with APH. The odds of neonatal mortality increased by 4.41 times [AOR = 4.41, 95%CI: (1.97,9.86)] for neonates born from mothers with pregnancy complicated with pregnancy-induced hypertension, 2.87 times [AOR = 2.87, 95% CI (1.08,7.61)] for neonates born from mothers with multiple pregnancy, and 5.05 times [AOR = 5.05, 95%CI: (1.72,14.79)] for neonates delivered at a health center.

The odds of death among newborns who had low birth weight were 4.01 times that of neonates who did not have the conditions [AOR = 4.01, 95%CI (1.30,12.33)]. Neonates with a diagnosis of perinatal asphyxia had 3.85 times higher odds of death compared to those neonates without perinatal asphyxia [AOR =3.85, 95%CI: (1.83,8.10)]. The odds of death among neonates who had early neonatal sepsis were 3.93 times that of neonates who did not have the conditions [AOR = 3.93, 95%CI: (1.84,8.41)], and the odds of death who had late neonatal sepsis were 4.59 times that of neonates who did not have the conditions [AOR = 4.59, 95%CI: (1.09,19.25)] (Table 3).

**Table 3:** Factors associated with neonatal mortality at Hiwot Fana Specialized University Hospital, Harar, Ethiopia, 2020

Variables		Neonatal mortality		COR (95% CI)	AOR (95%CI)
		Survived	Died		
ANC follow-up	Yes	544	73	1	1
	No	159	45	2.11 (1.398,3.182) **	0.864 (0.437,1.709)
APH	No	91	43	1	1
	Yes	38	43	2.395 (1.358,4.224) *	4.126 (1.923,8.853) **
PIH	No	91	45	1	1
	Yes	38	41	2.182 (1.237,3.85) *	4.406 (1.969,9.861) **
Type of pregnancy	Single	652	99	1	1
	Multiple	51	19	2.454 (1.391,4.328) *	2.869 (1.082,7.609) *
Place of delivery	Hospital	613	85	1	1
	Home	21	14	4.8 (2.356,9.811) **	3.475 (0.719,16.804)
	Health center	69	19	1.986 (1.139,3.46) *	5.048 (1.723,14.788) *
Prematurity	No	541	58	1	1
	Yes	162	60	3.455 (2.31,5.161) **	0.398 (0.127,1.244)
Low birth weight	No	496	43	1	1
	Yes	207	75	4.179 (2.778,6.29) **	4.005 (1.301,12.326) *
Sepsis	No	338	44	1	1
	Early-onset	290	62	1.642 (1.082,2.492) *	3.931 (1.837,8.412) **
	Late-onset	75	12	1.229 (0.619,2.440)	4.587 (1.093,19.252) *
Asphyxia	No	556	69	1	1
	Yes	147	49	2.686 (1.785,4.04) **	3.848 (1.829,8.096) **
Respiratory distress syndrome	No	680	103	1	1
	Yes	23	15	4.306 (2.175,8.52) **	1.662 (0.566,4.875)

*CI* Confidence Interval, *COR* Crude Odds Ratio, *AOR* Adjusted Odds ratio, *APH* Antepartum Hemorrhage, *MAS* Meconium Aspiration Syndrome, *RDS* Respiratory Distress Syndrome

*Significant with *P* < 0.05 and ** Significant with *P* < 0.001

## Discussion

In this study, it was attempted to indicate the magnitude of neonatal mortality. Majority of the neonatal death are due to preventable and treatable causes. Neonatal deaths mainly due to poor maternal health, inadequate care during pregnancy, inappropriate management of complications during pregnancy and delivery, poor hygiene during delivery and the first critical hours after birth, and lack of newborn care. The researchers tried to isolate factors that influence neonatal death at a tertiary hospital in Ethiopia. Accordingly, it has been observed that APH, PIH, type of pregnancy, place of delivery, LBW, asphyxia, and neonatal sepsis were factors significantly associated with neonatal mortality at NICU.

Certain evidence indicates that most of the Ethiopian hospitals NICU is not well equipped and higher patient- to nurse ratio which exposes the neonates to different complications that in turn increase the chance of dying [18]. The overall proportion of neonatal mortality in this study is consistent with studies conducted in Bangladesh 14.9% [22], Jimma 13.3% [21], and Gondar referral hospital, northwest Ethiopia 14.3% [4]. But the finding was lower compared to studies conducted in Ghana at 20.2% [20], MizanTepi University Teaching Hospital, southwest Ethiopia 22.8% [17], and Gondar referral hospital, northwest Ethiopia 23.1% [14]. This variation might be due to the lower sample size of the current study than studies [17, 20]. Another possible justification is that home delivery (10.1%) was high in Gondar compared to this study (4.3%) [14]. This may expose the neonate to infection, intrapartum, and postpartum complications because home delivery does not take place in aseptic conditions that have a deleterious effect on maternal and neonatal health.

However, the magnitude of neonatal mortality was higher than studies conducted in Iran at 10.23% [11], Cameroon at 10% [16], and Eritrea at 6.6% [1]. This discrepancy might be due to the health facility setups because some of the setups could be well-equipped, with the presence of skilled manpower. Another possible reason may be in this study about 26.2% of mothers of neonates had obstetrical complications during pregnancy compared to the study conducted in Eritrea (6.1%) [1]. So, the neonates may suffer different problems like Intra Uterine Growth Restriction (IUGR), asphyxia from pregnancy complications which lead to neonatal mortality.

In this study, Neonates born from mothers with current pregnancy complicated with antepartum hemorrhage (APH) was 4.13 times more likely to die as compared to neonates born from mothers without pregnancy complicated with APH. In fact that, pregnancy complicated with APH leads to fetal hypoxia then which leads to fetal bradycardia and fetal death [3]. Such complications can be reduced through the provision of proper antenatal care during pregnancy. Systematic review and meta-analysis study conducted in Ethiopia showed that a mother who had ANC follow-up had a positive impact in reducing neonatal mortality due to different obstetrical complications [23]. This implies that neonatal mortality due to APH can be decreased by utilization of antenatal care services.

Also, the odds of neonatal mortality for those neonates born from mothers with current pregnancy complicated with pregnancy-induced hypertension was 4.41 times that of neonates born from mothers without current pregnancy complicated with pregnancy-induced hypertension. The possible reason is that pregnancy-induced hypertension leads to uteroplacental dysfunction which results in nutritional and oxygen supply to fetus deficiency and causing IUGR, preterm delivery, and low birth weight which results in neonatal death [3]. According to UNICEF report, majority of the neonatal deaths are easily preventable with effective interventions administered during pregnancy and childbirth [25]. However, there is a certain degree of variation in the provision of health care services for the mothers and their newborns. In turn, mothers are exposed to different obstetrical complications that result in deterioration of the life of the mothers and newborns.

In this study, Neonates born from mothers with multiple pregnancy were 2.87 times higher odds of neonatal mortality than those of singleton pregnancy. The possible justification is that multiple pregnancy causes preterm delivery IUGFR, congenital malformation, and LBW [3]. These complications lead to neonatal death.

The odds of neonatal mortality among neonates delivered at the health center were 5.05 times that of neonates delivered at hospitals. This is consistent with studies conducted in Nigeria and Mauritania [19, 29]. This may be due to the unavailability of skilled manpower and equipment to treat those sick neonates immediately after delivery in the health center.

The majority of neonatal deaths in low- and mid-income countries are due to preventable and treatable conditions. A systematic review and meta-analysis done in Ethiopia show that most of the neonates died due to perinatal asphyxia, neonatal sepsis, prematurity problems, and birth injuries [23]. Health service utilization during pregnancy and child birth minimize neonatal death due to those preventable conditions, similarly in this study, the odds of death among newborns that had low birth weight were 4.01 times that of neonates who did not have the conditions. This is consistent with studies conducted in Brazil, Guinea-Bissau, Eritrea, Jimma, and eastern Ethiopia [1, 5, 9, 13, 21]. The possible justification is that in fact that low birth weight had immaturity of immune systems and, other body defense mechanisms that control newborn disease susceptibility [15]. Then the neonates may develop health problems including RDS, bleeding from the brain, NEC, and finally, neonatal death may follow.

Similarly, neonates with a diagnosis of perinatal asphyxia had 3.85 times higher odds of death compared to those neonates without birth asphyxia. This is consistent with studies conducted in Ghana, Gondar, Mekele, and Jimma, Ethiopia [4, 10, 14, 20]. The possible justification is that asphyxia causes carbon dioxide levels to increase and causing acidosis which leads to hypotension and ischemia then leads to brain cell injury then neonatal death [15].

Neonatal mortality remains an urgent concern of the country. A systematic review and meta-analysis conducted in Ethiopia showed that majority of the neonatal deaths that occur after delivery were due to neonatal sepsis because neonates are susceptible to infections and the progression of the disease are more rapid due to developmental immunodeficiency [36]. Similarly, in this study the odds of death among neonates who had early neonatal sepsis been 3.93 times that of neonates who did not have the conditions, and the odds of death which had late neonatal sepsis been 4.59 times that of neonates who did not have the conditions. This is consistent with studies conducted in Ghana, Gondar, and Mekele, Ethiopia [4, 10, 14, 20]. The possible justification is that sepsis results in abscess formation, venous thrombosis, neurologic damage, and multi-organ dysfunction [35]. Finally, neonatal mortality may follow. The death of neonates due to neonatal sepsis can be minimized by the provision of proper postnatal care and proper health service at NICU [31]. Evidence showed that neonates who get proper care at NICU had a lower chance of death due to sepsis [36]. Strengthening the care provided at the post-natal period and NICU is very important in reducing neonatal mortality.

### Limitation of study

The limitation of this study was it might not indicate a cause-effect relationship because the study design was cross-sectional. The use of medical records of newborns because of incompleteness and since the study is institution-based, the results might lack generalization to the entire population in the catchment area.

## Conclusion

The study revealed that the overall proportion of neonatal mortality was relatively in line with other studies conducted in Ethiopia but still needs attention. This study identified that pregnancy complications during current pregnancy like APH, and PIH, type of pregnancy, place of delivery, LBW, having PNA, and having neonatal sepsis were the independent factors associated with neonatal mortality. Most of the neonatal deaths are due to preventable and treatable conditions. Health care providers, hospital management should work hard to improve care for all neonates with special attention to the care of high-risk neonates and should focus on factors that affect neonatal survival to reduce neonatal mortality.

## Data Availability

The data set generated or analyzed during the current study are not publicly available due to the privacy of the participants and institution restriction but are available from the corresponding author on reasonable request.

## Abbreviations

ANC: Antenatal care
APGAR: Appearance Pulse Grimace Activity Respiration
APH: Antepartum Hemorrhage
HFSUH: Hiwot Fana Specialized University Hospital
NICU: Neonatal Intensive Care Unit
NMR: Neonatal Mortality Rate
UNICEF: United Nations Children’s Fund
WHO: World Health Organization
